# Genetic architecture of rind penetrometer resistance in two maize recombinant inbred line populations

**DOI:** 10.1186/1471-2229-14-152

**Published:** 2014-06-03

**Authors:** Kun Li, Jianbing Yan, Jiansheng Li, Xiaohong Yang

**Affiliations:** 1 National Maize Improvement Center of China, Beijing Key Laboratory of Crop Genetic Improvement, China Agricultural University, Beijing 100193, China; 2 National Key Laboratory of Crop Genetic Improvement, Huazhong Agricultural University, Wuhan 430070, China

**Keywords:** Maize, Rind penetrometer resistance, QTL, SNP

## Abstract

**Background:**

Maize (*Zea Mays* L.) is one of the most important cereal crops worldwide and provides food for billions of people. Stalk lodging can greatly undermine the standability of maize plants and therefore decrease crop yields. Rind penetrometer resistance is an effective and reliable method for evaluating maize stalk strength, which is highly correlated with stalk lodging resistance. In this study, two recombinant inbred line populations were constructed from crosses between the H127R and Chang7-2 lines, and between the B73 and By804 lines. We genotyped these two populations and their parents using 3,072 single nucleotide polymorphism markers and performed phenotypic assessment of rind penetrometer resistance in multiple environments to dissect the genetic architecture of rind penetrometer resistance in maize.

**Results:**

Based on two linkage maps of 1,397.1 and 1,600.4 cM with average interval of 1.7 and 2.1 cM between adjacent makers, respectively, seven quantitative trait loci (QTL) for rind penetrometer resistance were detected in the two recombinant inbred line populations. These QTL were distributed in seven genomic regions, and each accounted for 4.4–18.9% of the rind penetrometer resistance variation. The QTL with the largest effect on rind penetrometer resistance, *qRPR3-1*, was located on chromosome 3 with the flanking markers PZE-103123325 and SYN23245. This locus was further narrowed down to a 3.1-Mb interval by haplotype analysis using high-density markers in the target region. Within this interval, four genes associated with the biosynthesis of cell wall components were considered as potential candidate genes for the rind penetrometer resistance effect.

**Conclusions:**

The inheritance of rind penetrometer resistance is rather complex. A few large-effect quantitative trait loci, together with a several minor-effect QTL, contributed to the phenotypic variation in rind penetrometer resistance in the two recombinant inbred line populations that were examined. A potential approach for improving stalk strength and crop yields in commercial maize lines may be to introgress favorable alleles of the locus that was found to have the largest effect on rind penetrometer resistance (*qRPR3-1*).

## Background

Plant lodging is a complicated phenomenon that is affected by several factors, including genetics, environment and field management. Lodging is a considerable challenge for main crops during the growth as it often causes severe reduction in yields. In maize (*Zea Mays* L.), stalk lodging, breakage that occurs at or below the ear, can lead to loss of ears at harvest [1,2]. It is estimated that yield losses caused by stalk lodging range from 5 to 20% worldwide [1,3]. Additionally, stalk lodging poses an obstacle to mechanized harvesting, and consequently increases labor costs. Thus, improving stalk-lodging resistance has become a key target for maize breeding programs.

Developing an effective and accurate way to evaluate stalk-lodging resistance is a critical issue in improving maize stalk strength. Numerous quantitative methods have been developed to predict stalk lodging resistance potential, which mainly include chemical methods based on analysis of stalk chemical composition and anatomical structures, and mechanical methods based on measurements of stalk breaking, bending, penetration and crushing [4-9]. Among the mechanical methods, rind thickness and crushing strength have been useful in increasing lodging resistance in maize as they have shown a strong relationship with stalk lodging [7,9]. However, these two methods are not ideal because they require the destruction of maize stalk. More recently, an efficient and non-destructive measure, rind penetrometer resistance (RPR), was developed to assess stalk strength [10]. Increased RPR shows a high correlation with stalk-lodging resistance [11-15] and this method has been widely applied in estimating stalk lodging resistance potential in maize [2,16-18] and in breeding maize hybrids that are highly resistant to stalk lodging [1,11,12,19,20].

Despite these advances in measuring stalk lodging, little was known about the genetic basis of stalk lodging and RPR variation until quantitative trait loci (QTL) mapping was applied to RPR. The first of these studies identified 35 individual QTL and 11 pairs of epistatic interactions associated with RPR in four F_2:3_ populations derived from B73, Mo47 and four inbred maize lines selected for stalk strength diversity [2]. The majority of these QTL explained <15% of the phenotypic variation in RPR. An additional nine individual QTL and one more pair of epistatic interactions were detected in a recombinant inbred line (RIL) population crossed with a high-oil inbred line and B73, which account for another 1.15–12.43% of the phenotypic variation [17]. Recently, 18 QTL and 141 significant associations for RPR were identified using a nested association mapping panel containing 4,536 lines and 174 intermated B73 × Mo17 RILs. Only 10 QTL were shared between two populations or two studies, reflecting the complex nature of stalk lodging [2,17,18].

The availability of the complete maize genome sequence [21] and haplotype maps [22,23] have facilitated the development of single nucleotide polymorphism (SNP) genotyping technologies for maize [24]. SNPs have become widely used markers in investigations of genetic variation and in linkage and association analyses in maize [25-32]. Compared with simple sequence repeat markers, SNPs are more accurate, less time-consuming and lower cost to identify, and better suited to high throughput genotyping platforms [33-35]. Currently, two GoldenGate assays containing 1,536 SNPs each and one Infinium BeadChip containing 56,110 SNPs have been developed for maize [34,36,37]. These SNP assays have been successfully used to examine the population structure and estimate genetic diversity of maize populations [34,36,37], and to identify variants associated with maize agronomic and quality traits [25-32].

In this study, we developed two RIL populations, H127R × Chang7-2 (referred to as POP-HRC) and B73 × By804 (referred to as POP-BYB), from four inbred lines with varying stalk strengths, and genotyped them using a GoldenGate maize SNP assay containing 3,072 SNPs to increase QTL resolution. Our objectives were to (1) identify QTL associated with RPR of maize stalks; (2) dissect the main-effect QTL with detailed haplotype in the target region; and (3) mine candidate genes associated with maize stalk strength.

## Results

### Phenotypic variation in RPR

**Figure 1:**
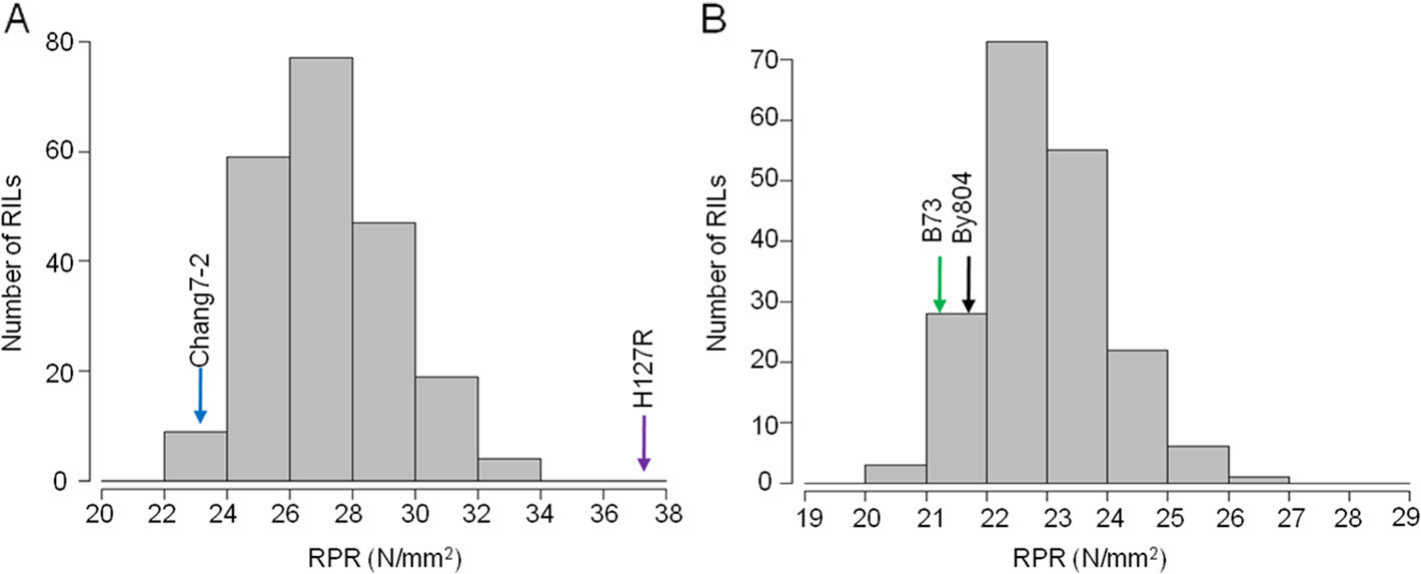
**Frequency distribution of RPR for RILs in the two populations in all environments. (A)** POP-HRC. **(B)** POP–BYB. Parental strain values are indicated with arrows.

Significant difference in RPR was observed between the H127R and Chang7-2 parental lines, whereas no significant difference was observed between the B73 and By804 lines (Table 1). Among these four parental lines, H127R, which is highly resistant to stalk lodging, had the highest RPR (37.64 ± 6.18 N/mm^2^), followed by Chang7-2 (23.25 ± 2.21 N/mm^2^), By804 (21.67 ± 2.63 N/mm^2^) and B73 (21.09 ± 2.82 N/mm^2^). The mean RPR value for the H127R × Chang7-2 RIL population (hereafter referred to as POP-HRC) was lower than the mean parent value, and the mean RPR value for the B73 × By804 RIL population (hereafter referred to as POP-BYB) was higher than the mean parent value (Table 1). RPR in both RIL populations showed a wide range with a normal distribution (Figure 1). Highly significant effects of genotype, environment, and genotype × environment interactions on RPR were observed in POP-HRC. In POP-BYB, both genotype and environment showed significant effects on RPR (Table 1). Broad-sense heritability estimates were 81.5% and 74.6% for POP-HRC and POP-BYB, respectively (Table 1).

**Table 1:** Descriptive statistics and broad-sense heritability for RPR in two RIL populations

**Population**	**POP-HRC**		**POP-BYB**	
Parents				
Means ± SD (N/mm^2^)	H127R	37.64 ± 6.18	By804	21.67 ± 2.63
	Chang7-2	23.25 ± 2.21	B73	21.09 ± 2.82
RILs				
Population mean ± SD (N/mm^2^)	27.19 ± 2.12		22.95 ± 1.03	
Range (N/mm^2^)	22.59–33.65		20.75–26.13	
F value Environment	397.20**		116.43**	
Genotype	9.51**		3.94**	
Environment × Genotype	1.76**			
Replication (environment)	3.75			
Heritability (*H*^2^) ^a^ (%)	81.5		74.6	
Confidence interval^b^	77.5–84.8		69.2–78.8	

**Significant at P < 0.01.

^a^Broad-sense heritability (*H*^2^) of RPR.

^b^90% confidence intervals of broad-sense heritability.

### Summary of SNPs and genetic linkage maps

A total of 2,866 and 3,029 high-quality SNPs were detected for POP-HRC and POP-BYB, respectively. The missing rate for these SNPs ranged from 0 to 15.35% in POP-HRC (average 0.83%) and from 0 to 17.02% in POP-BYB (average 0.90%). The minor allele frequency (MAF) for these SNPs ranged from 0 to 0.5 in both populations, with an average of 0.21 in POP-HRC and 0.23 in POP-BYB and the heterozygosity ranged from 0 to 13.49% in POP-HRC (average 2.38%) and from 0 to 4.79% in POP-BYB (average 0.92%) (Table 2). 6% (12/200) RILs of POP-BYB with SNP heterozygosity ≥ 0.1 were excluded for further analysis. Of the high-quality SNPs, 2,252 SNPs were polymorphic in one or both of the RIL populations. 1,391 SNPs (45.3%) were polymorphic between the parents of POP-HRC, 1578 SNPs (51.4%) were polymorphic between the parents of POP-BYB, and 717 SNPs (23.3%) were polymorphic in both RIL populations.

**Table 2:** Summary of SNP characteristics in the two RIL populations

	**POP-HRC**		**POP-BYB**	
	**Mean ± SD**	**Range**	**Mean ± SD**	**Range**
High-quality SNP number	2866		3029	
SNP missing rate (%)	0.83 ± 1.44	0.00–15.35	0.90 ± 1.60	0.00–17.02
MAF of SNPs	0.21 ± 0.22	0.00–0.50	0.23 ± 0.22	0.00–0.50
SNP heterozygosity (%)	2.38 ± 2.86	0.00–13.49	0.92 ± 1.10	0.00–4.79
SNP missing rate in each line (%)	0.83 ± 0.53	0.07–3.49	0.90 ± 2.46	0.00–17.70
SNP heterozygosity in each line (%)	2.40 ± 1.48	0.10–7.68	0.92 ± 1.12	0.00–5.88

After quality control, 822 SNPs in POP-HRC (215 lines), and 756 SNPs in POP-BYB (188 lines) were used to construct linkage maps. The total length of the linkage map for POP-HRC was 1,397.1 cM with an average interval of 1.7 cM, and the total length of the map for POP-BYB was 1,600.4 cM with an average interval of 2.1 cM (Table 3, Additional file 1). The relative locations of 86.3% (710) of the SNPs in POP-HRC and 68.1% (515) of the SNPs in POP-BYB were the same as their physical locations in the B73 reference genome Version 5b.60 [38]. Among these mapped SNPs, 37.0% (304) in POP-HRC and 24.9% (188) in POP-BYB showed segregation distortion at P < 0.05, which formed nine and six hot blocks of segregation distortion, respectively (Additional file 1).

**Table 3:** Summary of the linkage map characteristics of the two RIL populations

	**POP-HRC**					**POP-BYB**				
**Chromosome**	**Number of markers**	**Length (cM)**	**Average interval (cM)**	**Minimum interval (cM)**	**Maximum interval (cM)**	**Number of markers**	**Length (cM)**	**Average interval (cM)**	**Minimum interval (cM)**	**Maximum interval (cM)**
1	99	182.4	1.8	0.2	7.5	117	253.1	2.2	0.3	13.3
2	96	162.2	1.7	0.2	14.0	74	178.5	2.4	0.3	15.4
3	80	171.6	2.2	0.2	9.7	60	184.4	3.1	0.3	14.3
4	88	155.8	1.8	0.2	11.5	84	141.0	1.7	0.3	8.2
5	95	168.0	1.8	0.3	8.7	91	171.2	1.9	0.3	14.6
6	75	117.9	1.6	0.2	8.3	83	148.2	1.8	0.3	15.0
7	73	131.8	1.8	0.2	11.8	90	172.5	1.9	0.3	12.0
8	87	111.7	1.3	0.2	13.2	73	140.5	1.9	0.3	11.9
9	81	111.4	1.4	0.1	13.1	50	125.7	2.5	0.3	11.4
10	48	84.3	1.8	0.2	12.3	34	85.3	2.5	0.3	14.2
All	822	1,397.1	1.7			756	1,600.4	2.1		

### QTL analysis

In total, seven QTL were detected that appeared to be associated with RPR in the two RIL populations (Table 4, Additional file 1). The empirical threshold logarithm of odds (LOD) values for the genome-wide significance (P < 0.05) were determined to be 3.1 for POP-HRC and 3.2 for POP-BYB after 1000 permutations. These seven QTL were distributed in seven genomic regions across five chromosomes with marker intervals ranging from 0.6 to 24.9 Mb. The phenotypic variation in RPR explained by each QTL ranged from 4.4% (*qPPR2*) to 18.9% (*qPPR3-1*). We identified three main-effect QTL in three chromosomal regions which accounted for >10% of the RPR variation.

**Table 4:** RPR-associated QTL in the two RIL populations

**Population**	**QTL**	**Chromosome**	**Peak**^**a **^**(cM)**	**Marker interval**	**Genetic interval (cM)**	**Physical position**^**b **^**(Mb)**	**LOD**	**A**^**c**^	**R**^**2 **^**(%)**^**d**^
POP-HRC	*qRPR2*	2	162.1	SYN6917–PZE102193611	160.1-162.2	236.4–237.0	3.8	0.45	4.4
	*qRPR3-1*	3	107.4	PZE-103123325–SYN23245	104.5-111.1	181.1–184.7	14.0	1.05	18.9
	*qRPR3-2*	3	133.9	PZE-103156977–PZE-103160158	132.4-134.2	209.1–211.2	5.9	0.61	6.7
	*qRPR9*	9	47.0	PZE-109058177–PZE-109076761	42.4-50.0	99.4–124.3	6.6	0.66	8.1
	Total^e^								50.4
POP-BYB	*qRPR4*	4	55.7	PZE-104080388–PZE-104084757	50.3-55.7	154.7–158.7	7.9	-0.39	14.0
	*qRPR6-1*	6	89.4	PZE-106088503–SYN4646	88.5-91.9	146.1–147.7	3.6	0.27	6.0
	*qRPR6-2*	6	143.3	SYN34377–PHM3466.69	133.3-148.2	163.2–167.0	6.2	-0.39	13.8
	Total^e^								31.7

^a^The peak position with the highest LOD of each QTL.

^b^The physical positions of the identified QTL according to B73 reference sequence Version 5.60 [38].

^c^ Additive effect of the identified QTL: a positive value indicates that the alleles from H127R and By804 increases RPR, and a negative value indicates that the alleles from Chang7-2 and B73 increase RPR.

^d^Percentage of phenotypic variation explained by additive effects of the identified QTL.

^e^Total percentage of phenotypic variation explained by all QTL computed by MIM.

In POP-HRC, four of the RPR-associated QTL, located on chromosomes 2, 3 and 9 (Table 4, Additional file 1), together explained 50.4% of the phenotypic variation. The QTL on chromosome 3 flanked by the PZE-103123325 and SYN23245 markers, *qRPR3-1*, had the largest effect and accounted for 18.9% of the phenotypic variation in RPR. The H127R allele at this locus was correlated with a 1.05 N/mm^2^ increase in RPR. The second largest-effect QTL for RPR, *qRPR9*, which explained 8.1% of the phenotypic variation, was located between PZE-109058177 and PZE-109076761 on chromosome 9. The remaining two QTL, *qRPR2* on chromosome 2 and *qPPR3-2* on chromosome 3, explained 4.4% and 6.7% of the phenotypic variation, respectively. The alleles that were associated with increased RPR at these two loci also came from H127R.

In POP-BYB, the remaining three RPR-associated QTL, *qRPR4, qRPR6-1 and qRPR6-2*, accounted for 31.7% of the phenotypic variation. The B73 alleles at *qRPR4* and *qRPR6-2* were correlated with similar 0.39 N/mm^2^ increases in RPR. The By804 allele at *qRPR6-1*, had an additive effect of 0.27 N/mm^2^ for increased RPR.

To further confirm the seven RPR-associated QTL identified using the best linear unbiased prediction (BLUP) values, we also mapped RPR-associated QTL in the RIL populations grown in different environments and replications grown in the same environments (Additional file 2). The association with RPR was stable for *qRPR3-1* in all environments/replications, whereas the remaining six QTL differed significantly in at least two environments/replications or showed obvious LOD peaks in different environments/replications. In addition to the original seven QTL, 13 QTL were identified that associated with RPR in one or two environments/replications.

Beyond individual QTL, one pair of epistatic QTL between *qRPR3-1* and *qRPR3-2* was detected in POP-HRC. The type of epistasis between *qRPR3-1* and *qRPR3-2* was additive interacted by additive. This pair of epistatic QTL explains 2.5% of the phenotypic variation with a positive effect on RPR coming from the parental digenic combination. None epistatic QTL were identified in POP-BYB.

### Fine mapping of *qRPR3-1* in POP-HRC

**Figure 2:**
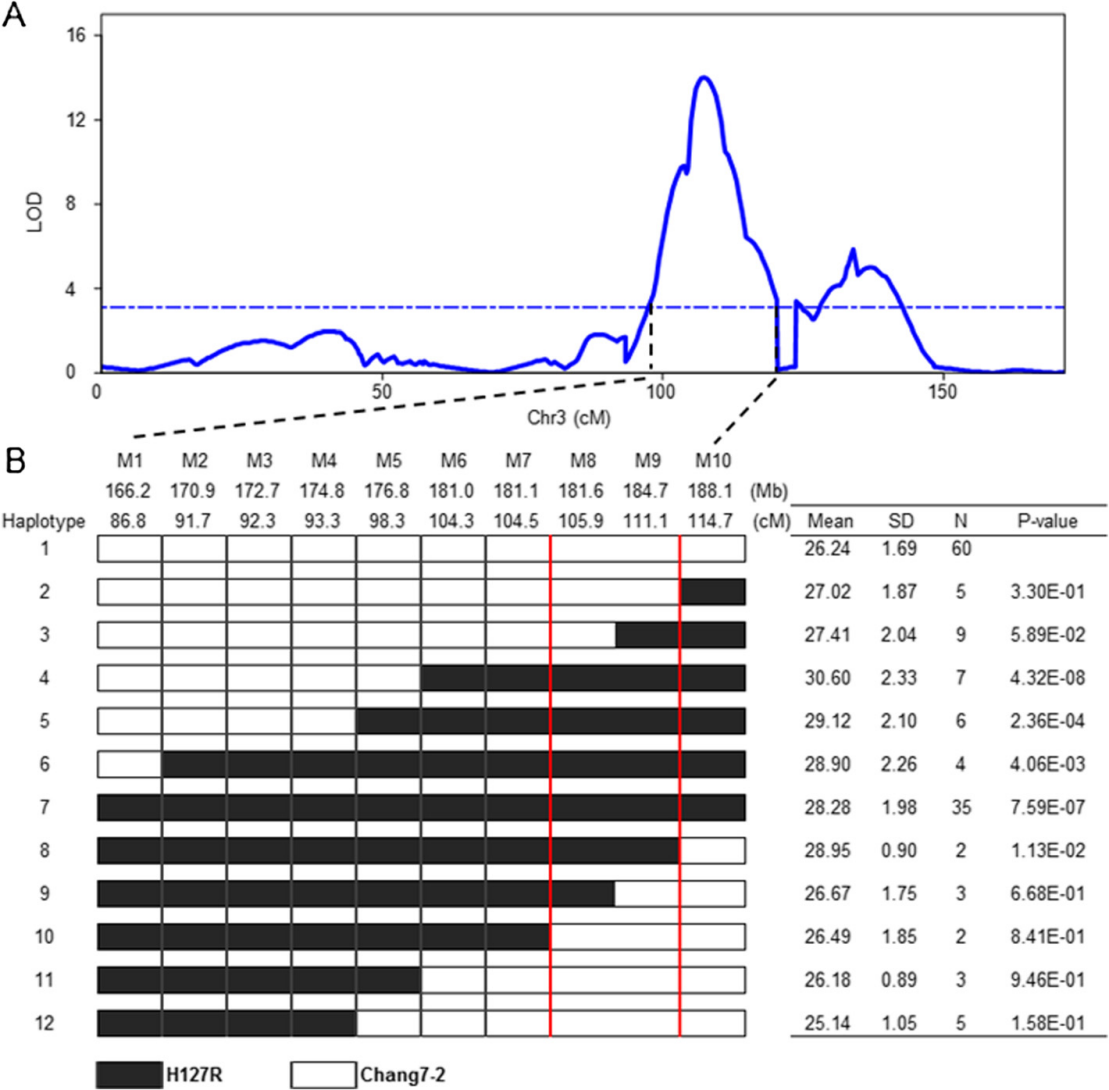
**Haplotype analysis and fine mapping of *qRPR3-1 *in POP-HRC. (A)** LOD profile for *qRPR3-1* estimated using the BLUP values of plants grown in the three locations/years. **(B)** Detailed haplotype analysis of the putative RPR-associated interval with the BLUP value of RPR. The red lines indicate the narrowed interval of *qRPR3-1*, M1–M10 represent the SNP markers PZE-103104806, PZE-103110761, PZE-103112971, PZE-103114860, PZE-103118170, SYN31220, PZE-103123325, PZE-103123992, SYN23245 and PZE-103132112, respectively.

Because of the large effect of *qRPR3-1* and the high density of SNP markers available at this locus, we were able to precisely determine the critical recombination breakpoint using SNPs that were polymorphic between two parents of POP-HRC in the QTL interval. Initially, *qRPR3-1* was localized to between the SNP makers PZE-103104806 (M1) and PZE-103132112 (M10), with the LOD values of all SNP markers in this interval greater than 3.1 (Figure 2A). This region spanned a genetic distance of 27.9 cM, corresponding to a physical distance of 21.9 Mb in the B73 reference sequence Version 5b.60 [38]. Using 10 polymorphic SNP markers in this region, 20 haplotypes were observed for the 215 RILs in POP-HRC. Of these haplotypes, only 12 had only one recombination breakpoint in the QTL interval (Figure 2B). We next compared the mean RPR, estimated using the BLUP values, of individuals with and without H127R alleles using a two-sample *t*-test. We observed that the RPR values of haplotypes 4–8 (28.28–30.60 N/mm^2^) were significantly higher than the RPR value of haplotype 1 (26.24 N/mm^2^), which did not carry H127R alleles at any of the 10 SNPs (α = 0.05, P = 1.13 × 10^-2^–4.32 × 10^-8^), whereas haplotypes 2, 3, and 9–12 showed similar RPR values (25.14–27.41 N/mm^2^) to haplotype 1. Therefore, we were able to narrow the location of *qRPR3-1* to a 3.1-Mb window between the markers PZE-103123992 (M8) and SYN23245 (M9). To further confirm the interval narrowed down, we also performed haplotype analysis using the RPR value in each environment, and the identity interval was inferred (data unpublished).

### Candidate genes in the target QTL region

**Figure 3:**
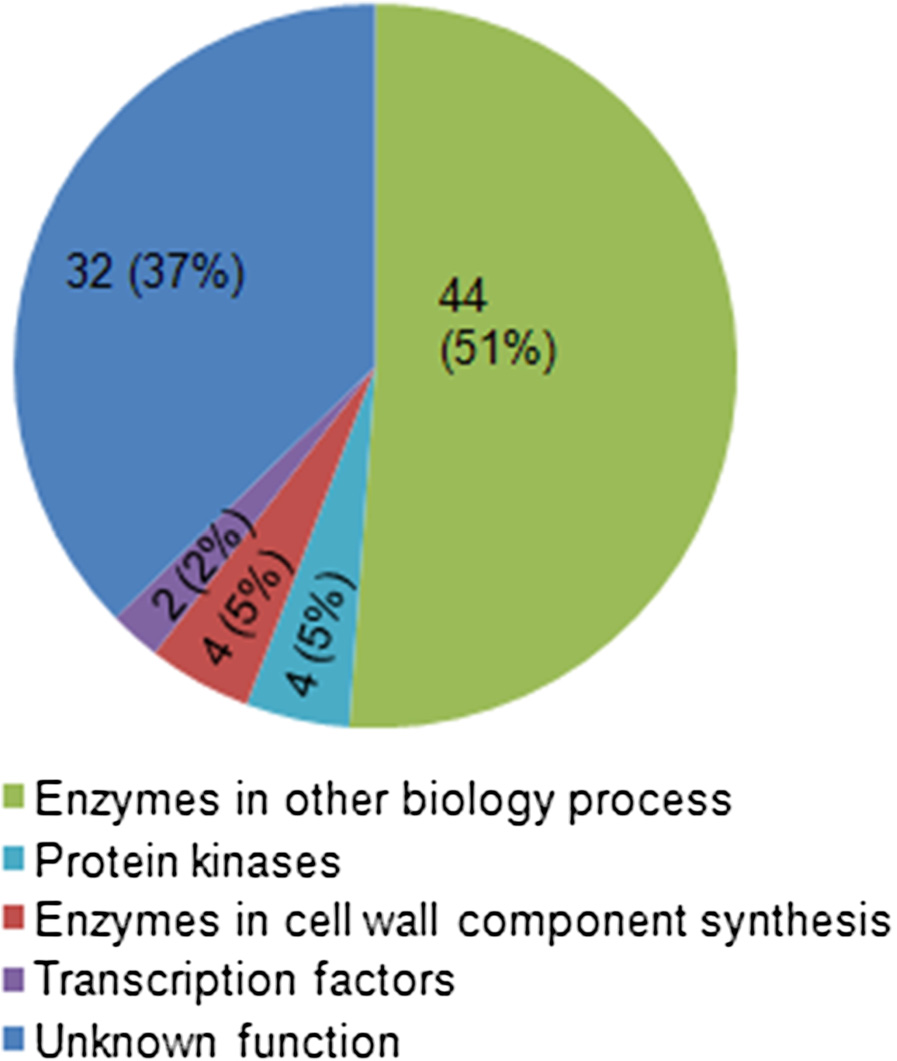
**Functional category annotations and respective percentages for 86 candidate genes within the narrowed *qRPR3-1 *** **interval.**

Based on the available annotation of the B73 reference sequence Version 5b.60 [38], there are 86 predicted genes in the 3.1-Mb target region (Additional file 3). Of these genes, 32 encode proteins of unknown function and the remaining 54 encode proteins that could be classified into four categories (Figure 3); protein kinases, enzymes involved in cell wall component synthesis and degradation, transcription factors, and enzymes related to other biological pathways.

## Discussion

### Genetic characterization of RPR in maize

Precise phenotypic measures are crucial for genotype-phenotype association analysis [39]. Previous studies have shown that RPR is highly associated with stalk-lodging resistance in maize [8,11,12,20]. For example, divergent selection for stalk crushing strength in synthetic maize populations has resulted in increased RPR [6,40]. In addition, RPR of the internodes below the uppermost ear has been found to be highly correlated with the RPR of internodes between the last ear and ground on maize plants [41]. Thus, measuring RPR of the internode below the uppermost ear is suggested to be one of the best ways to evaluate stalk-lodging resistance in maize in the current status.

Although RPR is a complex quantitative trait that can be affected by environment, most of the phenotypic variation appears to be due to genetic factors. The broad-sense heritability of RPR in maize, estimated in two previous studies as well as our study, reached over 90% in some segregating populations [2,17]. The high broad-sense heritability reflects the accuracy and feasibility of the method used to quantify RPR in these studies. Whereas, broad-sense heritability values of RPR in maize estimated from nested association map families are far lower than the values we estimated, ranging from 8 to 34% (averaged 21%) across 26 RIL populations [18]. The reduced heritability values may be attributable to the different populations surveyed, differences in the growing environments, or to the relatively low number of replications examined for each line in their study [18]. Further characterization of RPR in more bi-parent segregating populations is needed to reconcile these differences in heritability values.

### The complex nature of RPR in maize

The present study identified seven RPR-associated QTL were identified in two RIL populations. Among these QTL, only the largest-effect QTL, *qRPR3-1*, was also identified in two previous studies by Flint-Garcia et al. [2] and Hu et al. [17]; *qRPR3-2* and *qRPR6-2* were also detected in the Flint-Garcia et al. study and were found to explain 6.7% and 13.8% of the phenotypic variation, respectively [2]. Our study revealed that a few large-effect QTL, together with some minor-effect QTL, provide most of the genetic basis of RPR, consistent with previous studies [2,17,18]. Together with this study, a total of 69 RPR-associated QTL have been identified in 33 segregating populations. The phenotypic variation explained by the largest-effect QTL in each population ranged from 5.6 to 20.2%. Among these QTL, only ~10 were common in at least two populations. The low repeatability across populations may be due to the complex nature of RPR in maize, and the fact that most individual loci have small effects, which results in relatively small differences in RPR between parent strains [2,17,18].

In addition to single-effect QTL for RPR, Flint-Garcia et al. [2] and Hu et al. [17] detected 11 pairs of epistatic QTL in three F_2:3_ populations and one pair of epistatic QTL in one RIL population. The majority of these pairs of epistatic QTL explained <10% of phenotypic variations in each population. These findings, together with one pair of epistatic QTL identified in our study, suggest that epistasis also contributes to the genetic basis of RPR, but the effect of epistasis is relatively weak and not consistent in different bi-parent populations.

### Pleiotropic loci for stalk components

RPR is a physical measure of maize stalk strength, and significantly correlates with levels of some cell wall components [17,42]. Specifically, selection for stalk strength in a maize synthetic line using RPR found that divergent selection for increased RPR leads to increases in crude fiber, cellulose and lignin content in the plants [42]. Similarly, RPR significantly correlates with the levels of cell wall components, including acid detergent fiber, acid detergent lignin, crude fiber, cellulose, and neutral detergent fiber in internodes of a Ce03005/B73 RIL population [17]. The high correlation indicates that RPR-associated QTL may overlap, at least to some degree with QTL related to cell wall components of maize stalks.

Because RPR results from the combination of different cell wall component levels, it is likely that many of the same polymorphisms underlie the QTL associated with these two traits. Previous studies have identified 55 genomic regions related to cell wall components [43-45], including three loci in chromosome bins 3.06, 3.08 and 6.07 that are located near RPR-related genomic regions detected in our study. The largest-effect QTL for RPR in chromosome bin 3.06, *qRPR3-1*, co-localized with QTL clusters for acid detergent lignin and hemicelluloses detected in a F288 × F271 RIL × F286 top-cross population [45]. Another RPR-associated QTL in chromosome bin 3.08 in POP-HRC, *qRPR3-2*, is close to a QTL for Klason lignin in an RIL population derived from a cross between an old Minnesota13 line and a modern Iodent line [43]. This QTL also co-localizes with the hemicellulose-associated QTL in the F288 × F271 RIL × F286 top-cross population [45]. The POP-BYB QTL in chromosome bin 6.07, *qRPR6-2*, co-localized with a QTL that controls acid detergent fiber in De811 × B73 RIL populations [44]. Taken together, this information will help us to mine the candidate genes underlying QTL for RPR based on pleiotropy.

### Co-localization of RPR-related QTL and candidate genes

Generally, the final goal of primary QTL mapping is to clone genes of interest. Association analysis combined with traditional linkage analysis can speed up the process of cloning genes [46]. Recently, Xu et al. [32] reported that haplotype analysis using high-density markers within the target QTL interval in segregation populations is an effective way to narrow down the primary QTL with large effects. Applying this strategy, we narrowed *qRPR3-1* to a 3.1-Mb window predicted to contain 86 genes. Although this still leaves many possible genes within the loci that could be responsible for the RPR association of *qRPR3-1*, knowledge of connection between RPR and cell wall components can guide the choice of candidate genes for further study and potential cloning. Consequently, the four candidate genes known to be involved in cell wall component metabolism are considered the most likely to be the RPR-related *qRPR3-1* gene. *GRMZM2G132169* encodes a laccase that has been shown to play a role in lignin polymerization in *Arabidopsis thaliana*[47,48]. *GRMZM2G359234* encodes a UDP-glucuronic acid decarboxylase that produces UDP-xylose, a substrate for xylan biosynthesis [49], and decreased xylan content leads to reduced stem mechanical strength [50]. *GRMZM2G440016* encodes pectin methylesterase, which catalyses the de-esterification of pectin, and is significantly associated with stem strength in *Arabidopsis thaliana*[51]. *GRMZM2G126077* encodes the precursor of pectate lyase, and is involved in the pectin biodegradation pathway. Although some evidence from *Arabidopsis* supports the idea that *GRMZM2G359234* and *GRMZM2G440016* are the top candidates to be the RPR-associated genes, further investigation is necessary to confirm this connection, such as candidate-gene association mapping, fine mapping, and functional validation. Besides these four candidate genes, the other genes of unknown function or genes with function not linked to cell wall metabolism may also be the true variant for *qRPR3-1*.

### Application of RPR-related QTL to the improvement of maize stalk strength

Stalk strength is an important factor in breeding maize varieties to maintain grain yield. Phenotypic selection based on RPR has been successful in improving stalk strength in several maize synthetic populations [11,12,19,20,42,52,53]. Marker-assisted selection (MAS) is an alternative way to improve target traits [54-57], including disease resistance in maize [58]. Flint-Garcia et al. compared the efficiency of MAS and phenotypic selection for RPR, and found that MAS was more efficient [1]. Therefore, understanding the genetic architecture of stalk strength will enhance efforts to optimize stalk strength, and ultimately mitigate stalk lodging. The large-effect QTL for RPR, *qRPR3-1*, is a potential QTL for improving stalk strength via MAS. Additionally, *qRPR3-1* was narrowed to a relatively small QTL interval, which reduces the probability of linkage drag with deleterious alleles. The genetic effects of *qRPR3-1* associated with maize stalk cell wall components will provide additional phenotypic markers to guide the introgression of favorable alleles at the *qRPR3-1* locus.

## Conclusions

In this study, we mapped RPR-associated QTL in two RIL populations using medium SNP-density based linkage maps. We found that four QTL in POP-HRC and three in POP-BYB explained 50.4% and 31.7% of RPR variation, respectively. Only one of the seven QTL accounted for >15% of the RPR variations. These findings indicate that a few large-effect QTL and additional minor-effect QTL contribute to the phenotypic variation in RPR in the two RIL populations, reflecting the complex nature of stalk strength. The largest-effect QTL in chromosome bin 3.06 in POP-HRC, *qRPR3-1*, was narrowed to a 3.1-Mb interval by haplotype analysis using high-density markers in the target QTL interval. Within this interval, four genes associated with the biosynthesis of cell wall component were considered the most likely candidate genes for the *qRPR3-1* locus. This information will be valuable for introgressing favourable alleles of *qRPR3-1* into elite inbred lines to enhance stalk strength, and in turn mitigate stalk lodging.

## Methods

### Genetic materials

One maize F_9_ RIL population, consisting of 200 lines, was derived from a cross between the B73 and By804 lines. B73 is an elite inbred line derived from the Iowa Stiff Stalk Synthetic maize population. By804 is an inbred line developed from a Beijing high-oil population. Due to the high heterozygosity of 12 RILs in this population (>10%), only the remaining 188 lines were selected for subsequent analysis. Another F_6_ RIL population, containing 215 lines, was constructed by crossing the inbred lines H127R and Chang7-2. H127R is a parental line of the elite hybrid Zhongnongda 4, and Chang7-2 is the male parent line of the hybrid Zhengdan985. H127R is more resistant to stalk lodging than Chang7-2. For simplicity, we refer to the B73 × By804 RIL population as POP-BYB, and the H127R × Chang7-2 RIL population as POP-HRC.

### Field experiments and phenotyping

All 415 RILs, together with the four parent lines, were planted in a randomized complete block design from 2011 to 2013. For POP-HRC, two replications were planted in each of three environments, including Beijing in 2012 and in 2013 and Henan in 2013. For POP-BYB, one replication was planted in each of six environments, including Hainan in 2011 and in 2012, and Beijing, Henan, Chongqing and Yunnan in 2012. Each line was grown in a single 2.5 m row, rows were 0.67 m apart, and planting density was 45,000 plants/ha. The RPR of six randomly selected plants in each row was evaluated in the middle of the flat side of the internodes below the primary ear with an electronic penetrometer (AWOS-SL04, Aiwoshi Company, Hebei, China) at two weeks after flowering at the average level of each population, which roughly corresponded with the milk stage.

### Phenotypic data analysis

The variance components of RPR were estimated using PROC GLM in SAS 9.2 (SAS Institute). The model for variance analysis for POP-HRC was: *y*_*ijk*_ *= μ + e*_*l*_ *+ r*_*k(l)*_ *+ f*_*i*_ *+ (fe)*_*il*_ *+ ϵ*_*lik*_, where *μ* is the grand mean of RPR, *f*_*i*_ is the genetic effect of the “i”th line, *e*_*l*_ is environmental effect of the “l”th environment, *(fe)*_*il*_ is the interaction effect between genetic and environmental effects, *r*_*k(l)*_ is effect of replications within environments, and ϵ_*lik*_ is the residual error. For POP-BYB, the interaction effect between environment and genotype was treated as residual error due to the fact that there were no replications within each environment. These variance components were used to calculate broad-sense heritability based on the population means [59]. The broad-sense heritability in POP-HRC was estimated as $h^{2}=\sigma_{g}^{2}/\left(\sigma_{g}^{2}+\sigma_{\mathrm{ge}}^{2}/e+\sigma_{\epsilon }^{2}/\mathrm{re}\right)$, where $\sigma_{g}^{2}$ is the genetic variance, $\sigma_{\mathrm{ge}}^{2}$ is the interaction of genotype with environment, $\sigma_{\epsilon }^{2}$ is the residual error, *e* and *r* represent the number of environments and replications in each environment. In POP-BYB, the broad-sense heritability was estimated as $h^{2}=\sigma_{g}^{2}/\left(\sigma_{g}^{2}+\sigma_{\epsilon }^{2}/e\right)$, where $\sigma_{g}^{2}$ is the genetic variance, $\sigma_{\epsilon }^{2}$ is the residual error, *e* stands for the number of environments. Confidence interval of *h*^2^ were calculated according the method described by Knapp et al. [60].

A mixed linear model was fitted to each RIL to obtain the BLUP for RPR: *y*_*i*_ *= μ + f*_*i*_ *+ e*_*i*_ *+ ϵ*_*i*_, where *y*_*i*_ is the phenotypic value of individual i, *μ* is the grand mean for all environments, *f*_*i*_ is the genetic effect, *e*_*i*_ is effect of different environments, and *ϵ*_*i*_ is the random error. The grand mean was fitted as a fixed effect, and genotype and environment were considered random effects. The MIXED procedure in SAS9.2 (SAS Institute) was used to obtain the BLUP value.

### Genotyping and genetic map construction

Genomic DNA was extracted from leaf tissue of the RILs and parent lines using the modified CTAB method [61] and used for genotyping with the MaizeSNP3K subset (3,072 SNPs) of the Illumina MaizeSNP50 BeadChip [37]. SNP genotyping was performed on the Illumina GoldenGate SNP genotyping platform [62] at the National Maize Improvement Center of China, China Agricultural University. The quality of each SNP was checked manually as described by Yan et al. [34], and SNPs with poor quality were excluded for further analysis.

In each RIL population, the missing rate, MAF and heterozygosity for each SNP and the missing rate and heterozygosity for each line were calculated using PLINK packages [63]. The SNPs with missing rates ≤20% and MAFs ≥0.05 were used to construct the genetic linkage map with JoinMap 4.0 [64], using the Kosambi mapping function for calculating map distances. Linkage groups were formed at a minimum LOD of 6, and a regression-mapping algorithm was used to calculate map distances.

### QTL mapping

Windows QTL Cartographer 2.5 [65] was used for QTL detection with the RPR BLUP values across the different populations, environments and replications. The whole genome scan was performed using composite interval mapping with a 0.5 cM scanning interval between markers, and the window size was set at 10 cM. Model 6 of the Zmapqtl module was selected for detecting QTL and estimating their effects. Forward–backward stepwise regression with five controlling markers was used to control for background from flanking makers. After 1,000 permutations, the threshold LOD value was determined at a significance level of P < 0.05. The confidence interval of QTL position was determined with one-LOD support interval method [66]. To estimate the interactions of significant QTL and their total phenotypic variation, multiple interval mapping (MIM) in Windows QTL Cartographer 2.5 was performed with Bayesian Information Criteria (BIC-M0) as criteria of MIM model [67].

### Annotation of candidate genes

Based on the information available in the MaizeSequence database [38], the function of each gene within the largest-effect QTL interval was inferred from orthologues in Arabidopsis or rice. Additional protein prediction information was obtained from the InterPro module in the European Bioinformatics Institute database (http://www.ebi.ac.uk/interpro/) [68].

## Abbreviations

RPR: Rind penetrometer resistance; QTL: Quantitative trait loci; RIL: Recombinant inbred line; SNP: Single nucleotide polymorphism; cM: centimorgan; MAF: Minor allele frequency; POP-HRC: H127R/Chang7-2 population; POP-BYB: B73/By804 population; LOD: Logarithm of odds; MAS: Marker-assisted selection; BLUP: Best linear unbiased prediction.

## Competing interests

The authors declare that they have no competing interests.

## Authors’ contributions

LK carried out the experiments, analyzed data and wrote the manuscript; YJ carried out the field experiments; LJ designed the study and assisted in writing the manuscript; YX designed the study and wrote the manuscript. All authors read and approved the final manuscript.

## Supplementary Material

Additional file 1 **Genetic maps and distribution of putative RPR-related QTL in two RIL populations. (A)** POP-HRC. **(B)** POP-BYB. The red bar on each chromosome indicates the hot block of segregation distortion, and the black bar indicates the location of the identified QTL, the blue oval represents the centromere of each chromosome Click here for file

Additional file 2 **LOD profiles of the identified RPR-associated QTL in the RIL populations grown in different environments. (A)** POP-HRC. E1, 2013 Beijing replication 1; E2, 2013 Beijing replication 2; E3, 2013 Henan replication 1; E4, 2013 Henan replication 2; E5, 2012 Beijing replication 1; E6, 2012 Beijing replication 2; E7, BLUP. **(B)** POP-BYB. R1, 2011 Hainan; R2, 2012 Chongqing; R3, 2012 Yunnan; R4, 2012 Henan; R5, 2012 Beijing; R6, 2012 Hainan; R7, BLUP. Click here for file

Additional file 3 **Annotation of the 86 predicted genes located within the narrowed *qRPR3-1* interval in POP-HRC. ** Click here for file
