# Supplementary material for: Genetic architecture of rind penetrometer resistance in two maize recombinant inbred line populations
Source: BMC Plant Biol. 2014 Jun 3;14:152. doi: 10.1186/1471-2229-14-152 (PMC4053554; doi:10.1186/1471-2229-14-152)

###
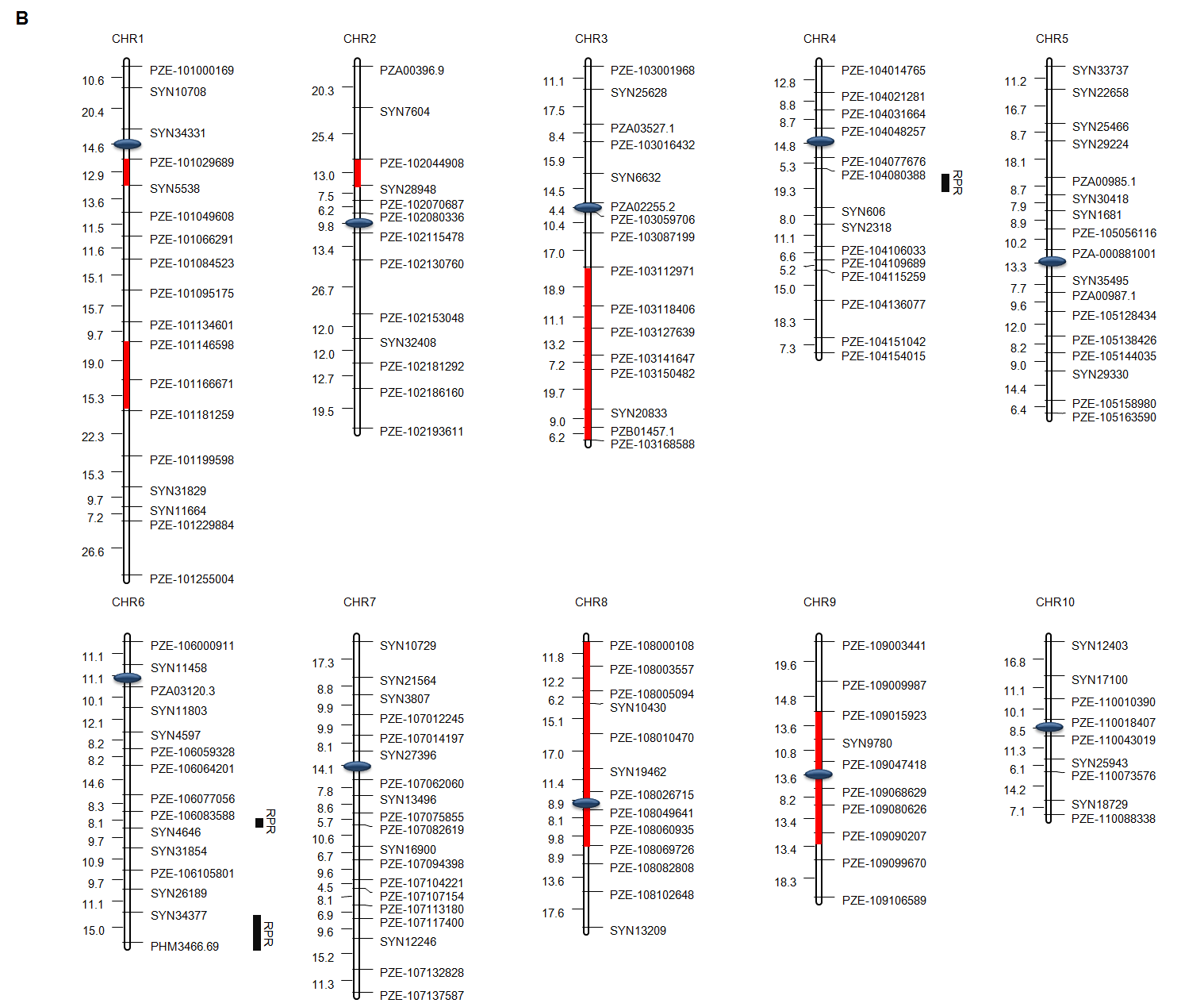
Additional file 1 –Genetic maps and distribution of putative RPR-related QTL in two RIL populations.(A) POP-HRC. (B) POP-BYB. The red bar on each chromosome indicates the hot block of segregation distortion, and the black bar indicates the location of the identified QTL, the blue oval represents the centromere of each chromosome.


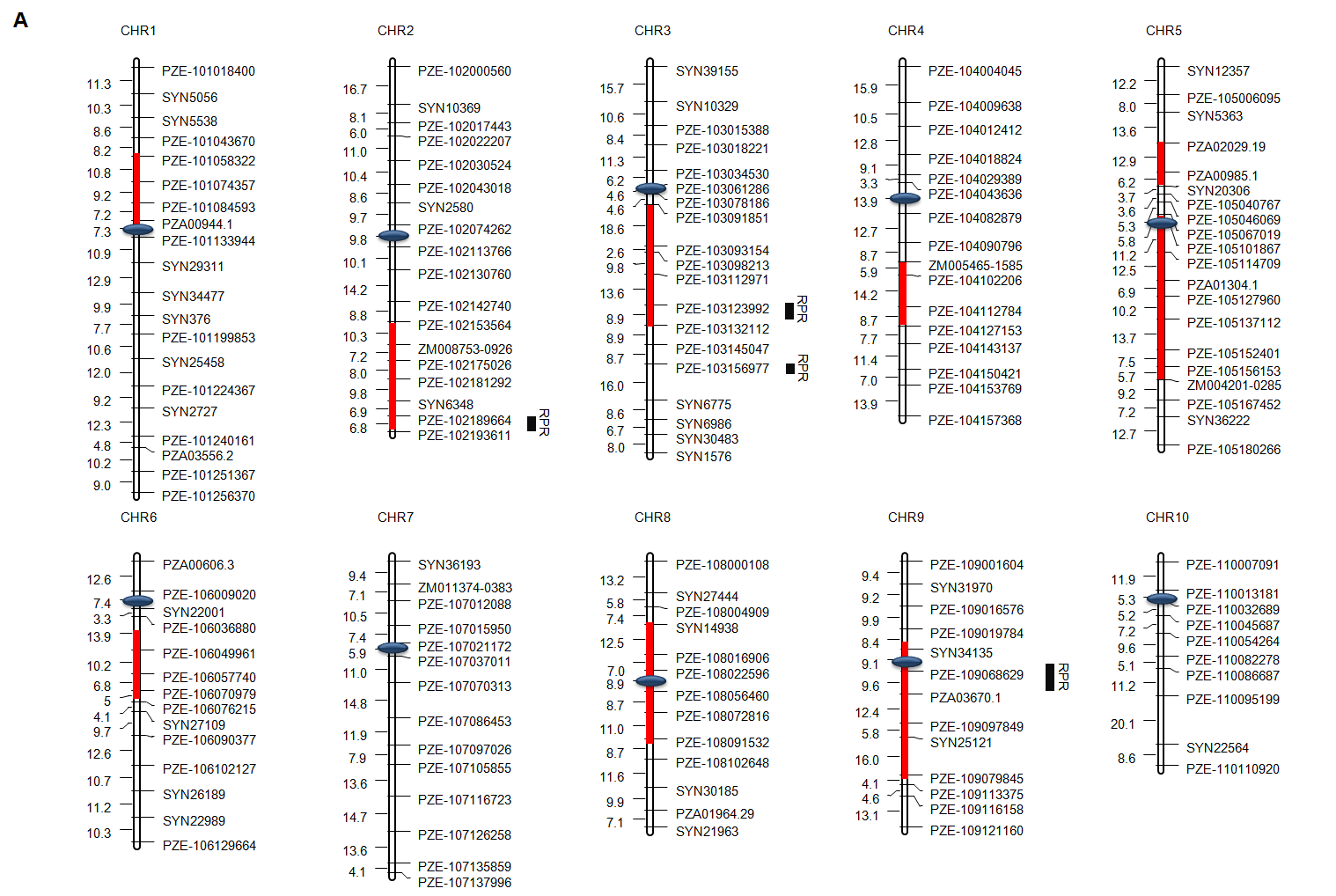

Supplement: Additional file 1 — Genetic maps and distribution of putative RPR-related QTL in two RIL populations. (A) POP-HRC. (B) POP-BYB. The red bar on each chromosome indicates the hot block of segregation distortion, and the black bar indicates the location of the identified QTL, the blue oval represents the centromere of each chromosome [file 1471-2229-14-152-S1.docx]
