# Supplementary material for: Genetic architecture of rind penetrometer resistance in two maize recombinant inbred line populations
Source: BMC Plant Biol. 2014 Jun 3;14:152. doi: 10.1186/1471-2229-14-152 (PMC4053554; doi:10.1186/1471-2229-14-152)

**Additional file 2 –LOD profiles of the identified RPR-associated QTL in the RIL populations grown in different environments.** (A) POP-HRC. E1, 2013 Beijing replicate 1; E2, 2013 Beijing replicate 2; E3, 2013 Henan replicate 1; E4, 2013 Henan replicate 2; E5, 2012 Beijing replicate 1; E6, 2012 Beijing replicate 2; E7, BLUP. (B) POP-BYB. R1, 2011 Hainan; R2, 2012 Chongqing; R3, 2012 Yunnan; R4, 2012 Henan; R5, 2012 Beijing ; R6, 2012 Hainan; R7, BLUP.


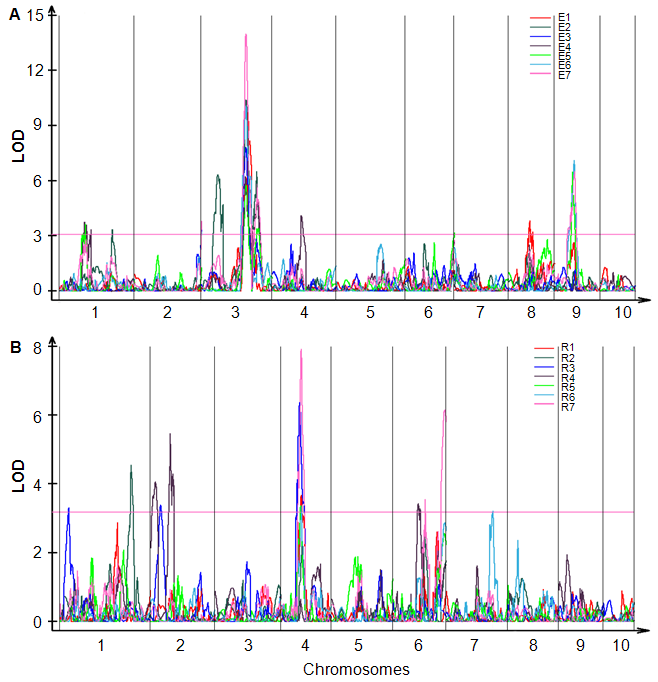

Supplement: Additional file 2 — LOD profiles of the identified RPR-associated QTL in the RIL populations grown in different environments. (A) POP-HRC. E1, 2013 Beijing replication 1; E2, 2013 Beijing replication 2; E3, 2013 Henan replication 1; E4, 2013 Henan replication 2; E5, 2012 Beijing replication 1; E6, 2012 Beijing replication 2; E7, BLUP. (B) POP-BYB. R1, 2011 Hainan; R2, 2012 Chongqing; R3, 2012 Yunnan; R4, 2012 Henan; R5, 2012 Beijing; R6, 2012 Hainan; R7, BLUP. [file 1471-2229-14-152-S2.doc]
