# Supplementary material for: Genetic architecture of rind penetrometer resistance in two maize recombinant inbred line populations
Source: BMC Plant Biol. 2014 Jun 3;14:152. doi: 10.1186/1471-2229-14-152 (PMC4053554; doi:10.1186/1471-2229-14-152)
Supplement: Additional file 3 — Annotation of the 86 predicted genes located within the narrowed qRPR3-1 interval in POP-HRC. [file 1471-2229-14-152-S3.doc]

### Additional file 3 –Annotation of the 86 predicted genes located within the narrowed *qRPR3-1* interval in POP-HRC.

| Gene ID | Start Position^a^ | End Position^a^ | Description |
| --- | --- | --- | --- |
| GRMZM2G302171 | 181616555 | 181617267 | Unknown |
| GRMZM2G386991 | 181650122 | 181654733 | Serine/threonine-protein kinase AFC1 /CDC-like kinase 3 |
| GRMZM2G014454 | 181679706 | 181688287 | Metal tolerance protein Mn-specific cation diffusion facilitator transporter |
| GRMZM2G059134 | 181690450 | 181696280 | Unknown |
| GRMZM2G359230 | 181698730 | 181700453 | Ruvb-1 encodes a AAA+ ATPase orthologous to the RUVBL1 family of ATPases. |
| GRMZM2G359234 | 181704344 | 181708313 | UDP-glucuronic acid decarboxylase 1 AT3G53520 |
| GRMZM2G126083 | 181748417 | 181750299 | Hydrolase activity |
| GRMZM2G126077 | 181751086 | 181752487 | Pectate lyase 4 precursor |
| GRMZM2G123977 | 181757576 | 181761519 | Ankyrin repeat-containing protein |
| GRMZM2G124016 | 181766089 | 181767509 | Unknown |
| AC214448.3_FG007 | 181790295 | 181791647 | Lung seven transmembrane receptor domain containing |
| GRMZM2G049893 | 181805648 | 181808646 | Unknown |
| GRMZM2G049877 | 181816685 | 181819465 | White-brown complex homolog protein |
| GRMZM2G349651 | 181825453 | 181828038 | H4 histone |
| GRMZM2G049510 | 181831769 | 181835657 | Unknown |
| GRMZM2G062218 | 181905273 | 181906809 | CCT motif family protein |
| GRMZM2G127040 | 182032506 | 182032995 | Unknown |
| GRMZM2G127034 | 182036583 | 182042328 | AT hook motif family protein |
| GRMZM2G426953 | 182041179 | 182046702 | Oxidation-reduction protein |
| GRMZM2G132614 | 182059192 | 182060511 | Unknown |
| GRMZM2G096470 | 182077112 | 182089424 | Rab GTPase activator activity |
| GRMZM2G326933 | 182126949 | 182127555 | Unknown |
| AC196780.4_FG003 | 182132070 | 182132336 | Unknown |
| GRMZM2G032258 | 182159627 | 182165670 | Zinc finger C-x8-C-x5-C-x3-H |
| GRMZM2G084465 | 182279648 | 182281549 | 40S ribosomal protein S23 |
| GRMZM2G303465 | 182417997 | 182420272 | Histone-like transcription factor and archaeal histone |
| GRMZM2G004314 | 182425971 | 182427118 | Vacuolar ATP synthase 98 kDa subunit |
| GRMZM2G023279 | 182483452 | 182488672 | Myosin heavy chain-related |
| GRMZM2G155954 | 182589300 | 182593175 | IQ calmodulin-binding motif domain containing protein |
| GRMZM2G456473 | 182595195 | 182596593 | Histone deacetylase |
| GRMZM2G033630 | 182709227 | 182713005 | OsSCP5 - Putative Serine Carboxypeptidase Homologue, expressed |
| GRMZM2G504401 | 182713971 | 182717776 | Hydrolase |
| GRMZM2G173542 | 182749400 | 182757534 | NLI interacting factor-like phosphatase |
| GRMZM2G475743 | 182752839 | 182754872 | Pentatricopeptide repeat |
| GRMZM2G448633 | 182776161 | 182781432 | Protein kinase family protein ATP binding protein |
| GRMZM2G122280 | 182849351 | 182854252 | Flavonol synthase/flavanone 3-hydroxylase |
| GRMZM2G014720 | 182896768 | 182900200 | BAG domain containing protein IPR003103 chaperone binding |
| GRMZM2G440016 | 182986035 | 182987807 | Pectinesterase |
| GRMZM2G440020 | 182988054 | 182988545 | Unknown |
| GRMZM2G302559 | 183036425 | 183041192 | Boron transporter protein |
| GRMZM2G397518 | 183084299 | 183085268 | Barren stalk1 |
| AC195348.3_FG004 | 183090121 | 183090453 | Unknown |
| GRMZM2G043095 | 183119585 | 183121793 | Leaf senescence related protein |
| GRMZM2G402618 | 183236038 | 183239409 | Unknown |
| GRMZM2G105283 | 183255168 | 183257231 | Unknown |
| GRMZM2G105307 | 183259255 | 183263602 | HIT zinc finger domain |
| GRMZM2G333726 | 183304206 | 183304828 | Gibberellin receptor GID1L2 |
| GRMZM2G032958 | 183311942 | 183313078 | Unknown |
| GRMZM2G032763 | 183323886 | 183325177 | Electron transporter glutaredoxin |
| GRMZM2G006229 | 183383001 | 183388746 | Thioredoxin |
| GRMZM2G034810 | 183441467 | 183442685 | Unknown |
| GRMZM2G045249 | 183485033 | 183490390 | Methyltransferase |
| GRMZM2G141350 | 183553178 | 183555430 | Dihydroflavonol-4-reductase |
| GRMZM2G141382 | 183556976 | 183560404 | Unknown |
| GRMZM2G141392 | 183573031 | 183574810 | Exocyst complex protein Exo70 |
| AC208346.3_FG004 | 183576733 | 183578676 | Exocyst complex protein Exo70 |
| GRMZM2G432671 | 183633434 | 183634975 | Unknown |
| GRMZM2G432662 | 183638286 | 183639680 | Prenylated Rab receptor 2 |
| GRMZM2G132169 | 183644921 | 183647436 | Laccases |
| GRMZM2G099080 | 183842979 | 183861125 | Unknown |
| GRMZM2G099036 | 183863367 | 183868467 | Unknown |
| GRMZM2G399218 | 183881116 | 183882083 | Unknown |
| GRMZM2G148087 | 183888339 | 183891343 | WRKY transcription factor |
| GRMZM2G011541 | 183998357 | 184001625 | Unknown |
| GRMZM2G118363 | 184059147 | 184065670 | Unknown |
| GRMZM2G118441 | 184068313 | 184070534 | Nodulin-like protein |
| GRMZM2G092517 | 184119671 | 184121779 | Unknown |
| GRMZM2G014979 | 184153351 | 184155241 | Unknown |
| GRMZM2G014945 | 184157482 | 184158411 | Unknown |
| GRMZM2G315769 | 184190721 | 184196613 | Serine/threonine transporter |
| GRMZM2G702036 | 184213947 | 184216375 | Unknown |
| GRMZM2G445201 | 184216449 | 184223082 | Unknown |
| AC193318.3_FG006 | 184263929 | 184264721 | Unknown |
| GRMZM2G027375 | 184268443 | 184270898 | Spotted leaf 11 |
| GRMZM2G027478 | 184278114 | 184282019 | Unknown |
| GRMZM2G330153 | 184279180 | 184280529 | DUF641 |
| GRMZM2G330159 | 184312959 | 184314337 | DUF623 |
| GRMZM2G339122 | 184363236 | 184365593 | Alpha expansin1 |
| GRMZM2G035726 | 184383282 | 184387046 | Peptidase C1A, papain C-terminal |
| GRMZM2G126397 | 184434264 | 184435351 | Nonspecific lipid-transfer protein |
| GRMZM5G852096 | 184460949 | 184462419 | Unknown |
| GRMZM2G426613 | 184461058 | 184462419 | RING-H2 finger protein |
| GRMZM2G461139 | 184605266 | 184606735 | Unknown |
| GRMZM2G068151 | 184627307 | 184630881 | Serine/threonine-protein kinase NAK |
| AC204009.3_FG009 | 184631674 | 184632285 | Unknown |
| GRMZM2G145753 | 184671622 | 184675445 | Receptor-like protein kinase precursor |

^a^Nucleotide position in the B73 reference sequence (version 5b.60; MaizeSequence, <http://www.maizesequence.org/>)
